# Supplementary material for: Developmental learning disorders in children with prenatal/perinatal exposure to hypoxia: A systematic review protocol
Source: PLoS One. 2023 Oct 20;18(10):e0293067. doi: 10.1371/journal.pone.0293067 (PMC10588827; doi:10.1371/journal.pone.0293067)
Supplement: S1 Checklist — (PDF) [file pone.0293067.s001.pdf]

PRISMA-P (Preferred Reporting Items for Systematic review and Meta-Analysis Protocols) 2015 checklist: recommended items to address in a systematic review protocol*

Section and topic	Item No	Checklist item
**ADMINISTRATIVE INFORMATION**		
Title:		
Identification	1a	Developmental learning disorder of children prenatally exposed to hypoxia: a protocol of systematic review.
Update	1b	NaN
Registration	2	In accordance with the guidelines, our systematic review protocol was registered with the International Prospective Register of Systematic Reviews (PROSPERO) on 7 December 2022 (registration number CRD42022371387).
Authors:		
Contact	3a	Author Affiliations; Bartosz M. Radtke 1*, Urszula Sajewicz-Radtke 1, Łucja Bieleninik 2,3, Małgorzata Lipowska 2<br>1 Laboratory of Psychological and Educational Tests, Gdańsk<br>2 Institute of Psychology, Faculty of Social Sciences, University of Gdansk, Gdańsk, Poland<br>3 GAMUT-The Grieg Academy Music Therapy Research Centre, NORCE Norwegian Research Centre, Bergen, Norway<br>*Correspondence: Bartosz M. Radtke radtke@pracowniatestow.pl
Contributions	3b	All authors were responsible and accountable for all parts of the work related to the protocol. More specifically, BMR had the original idea. BMR, ML and ŁB contributed to the conception and design of the protocol. GG performed the first literature searches. BMR, ML and USR contributed to writing the manuscript. All authors revised the manuscript and approved the final version to be published.
Amendments	4	If we need to amend this protocol, we will give the date of each amendment, describe the change, and give the rationale in this section. Changes will not be incorporated into the protocol.
Support:		
Sources	5a	This systematic review is funded by the "Perinatal risk factors for neurodevelopmental disorders in school-aged children." project, witch is carried out within the Polish Association of Educational Psychology (Nr. PTPE/2022/0482) and funds of the Laboratory of Psychological and Educational Tests.
Sponsor	5b	NaN
Role of sponsor or funder	5c	The funding body does not influence the design of the study and the writing of the manuscript.
**INTRODUCTION**		
Rationale	6	To the best of our knowledge, there is no systematic review nor meta-analysis on the association between exposure to prenatal/perinatal hypoxia and the occurrence of DLD symptoms at school age. Therefore, our motivation is to conduct the first systematic review of previous published observational studies (i.e., cohort studies, and case-control studies) that consider, as an exposure, presence of hypoxia during pregnancy or delivery and consider, as an outcome, DLD in the school-age children, as defined by the DSM-5 classification of neurodevelopmental outcomes. The understanding of the

		association between health-related event - hypoxia during pregnancy or delivery and outcome - DLD in school-age children will provide evidence in aetiology of DLD and thus, improve our understanding of the state of knowledge. The aim of this paper is to present the protocol for the aforementioned systematic review.
Objectives	7	This systematic review seeks to address the research question: Whether prenatal hypoxia is associated with developmental learning disorder in school age?
**METHODS**		
Eligibility criteria	8	Studies will be chosen based on the pre-specified below mentioned eligibility criteria.<br>**Population** (types of participants). The participants in the included studies will have been of primary school age, with no restrictions on sex or nationality, and have been diagnosed with DLD. DLD should have been diagnosed according to the national standards. Due to the research question of aetiology, we aim to exclude prematurely born children (defined as delivery up to 36;9 weeks of gestation).<br>**Exposure of interest** (independent variable). The review will include studies that consider, as an exposure, presence of prenatal/perinatal hypoxia indicated in the child's medical records.<br>**Comparator/Control**. We will include studies with participants of primary-school age, with no restrictions on nationality and without history of prenatal/perinatal hypoxia.<br>**Outcome** (dependent variable). The review will include studies that consider, as an outcome, presence of developmental learning disorder.<br>**Study Type**. We will include observational studies including cohort and case-control studies.<br>**Location**. We will not impose any restrictions on the location of the conducting of the research.
Information sources	9	We will search the National Medical Library, PsycINFO, Web of Science, EMBASE, DARE, and the Cochrane Library. Electronic database searching will be complemented by manual data searches. Reference lists of the included review articles will be checked to identify any additional studies. The search will be not restricted to any year of publication, sample size, or language (provided an English language translation of the abstract is available). We will exclude editorials and letters.
Search strategy	10	Medical Subject Headings (19) or equivalent and text word terms and words related to the nosological unit will be used to develop literature search strategies. Boolean operators and proximity operators (parentheses and quotations) will also be used. The search strategy will include terms relating to outcome (Learning Development Disorders [MeSH] OR Learning Disabilities OR dyslex* OR legasthenia OR learning disorder OR learning difficult* OR learning disabilit* OR LD OR RD OR SLD OR LRD OR DLD OR reading difficult* OR reading disabilit* OR reading impairment OR reading disorder* OR impairment in reading) and exposure of interest (hypoxia OR asphyxia OR oxygen deficiency OR oxygen shortage OR asphyxiation OR suffocation). The initial search strategy (including searching terms and filters) has already been piloted on PubMed in June 2023 to investigate whether it can find potentially relevant reports.
Study records:		
Data management	11a	One reviewer will search databases and handsearch the reference list of the included review articles. All potentially relevant records will be extracted to EndNote reference management software. At this stage, duplicates will be detected and deleted. After a year additional search will be done the same way.

Selection process	11b	Two authors independently will extract data from the studies based on a specifically designed data extraction form. Discrepancies will be resolved by discussion or/and consultation with third reviewer. Authors will be contacted in order to obtain any missing data or for clarification.
Data collection process	11c	Data will be extracted from the studies independently by two authors based on a purpose-designed pre-piloted data extraction form. This will be done by content area experts in the field of clinical psychology who are familiar with DLD and who will receive training in the coding of entries. Any discrepancies will be resolved by consultation/discussion with another reviewer. If a discrepancy cannot be resolved, we will contact by e-mail the study authors; if we are unsuccessful in doing so, we will report the discrepancies in the review. We will attempt to obtain any missing data from corresponding authors by e-mail. For different reports describing the same study/project, data will be extracted from each report separately and combined across multiple data collection forms afterwards.
Data items	12	The following information will be extracted from the studies:<br>• Characteristics of studies:<br>– first author, date of publication, DOI number, country of the study;<br>– study design (cohort vs. case-control).<br>• Characteristics of the respondents:<br>– age, sex;<br>– native language, languages spoken (mono vs. bilingualism); and<br>– years of education, intelligence level.<br>• Characteristics of Developmental Learning Disorder:<br>– type of DLD (formal diagnosis vs. poor readers);<br>– DLD subtype (dyslexia, dysorthography, reading and math problems);<br>– criteria of diagnosis (DSM, ICD, national standards);<br>– diagnosis provider (assessor, psychologist, psychiatrist, other), and<br>– comorbidities.<br>• Hypoxia characteristics:<br>– source of information about hypoxia (medical record vs. parental information);<br>– cause of hypoxia;<br>– methods of hypoxia treatment; and<br>– type of labor, APGAR score.<br>• Results<br>– effect sizes for the relation between hypoxia and dyslexia (e.g. Pearson's r for correlational analyses; semi-partial correlations or standardized beta coefficients for multivariate analyses)<br>– quantitative data of relation between hypoxia and dyslexia
Outcomes and prioritization	13	Outcome will be expressed as associations identified in the studies between prenatal hypoxia is associated with developmental learning disorder.<br>Odds ratios (ORs) and 95% confidence intervals (95%CIs) were used to assess the relationship between prenatal hypoxia is associated with developmental learning disorder.

Risk of bias in individual studies	14	The Newcastle–Ottawa Scale (NOS) will be used to assess the risk of bias in each included study. Two independent versions of NOS will be used to evaluate cohort and case-control studies. The NOS judge eight items, categorized into three domains including selection, comparability, and in respect to the study type - outcome (cohort studies) or exposure (case-control studies). Each study could receive a maximum of nine stars (one star for each numbered item within the selection and outcome categories, and a maximum of two stars for comparability). Two review authors will independently assess potential biases while any discrepancies will be resolved by discussion between reviewers; if necessary, content area experts from clinical trial methodology will be included in the discussion. Risk of bias will be categorized as "good", "fair", and "poor" for converting NOS assessments following guidelines of the Agency for Healthcare Research and Quality (AHRQ). Studies scored for 3 or 4 stars in the selection domain, 1 or 2 stars in the comparability domain, and 2 or 3 stars in the outcome/exposure domain will be considered to represent good quality. Studies rated 2 stars in the selection domain, 1 or 2 stars in the comparability domain, and 2 or 3 stars in the outcome/exposure domain were considered to represent fair quality. Papers evaluated as being 0 or 1 stars in the selection domain, 0 stars in the comparability domain, and 0 or 1 stars in the outcome/exposure domain were considered to be poor quality. We don't plan to evaluate an overall quality of evidence since there are no plans for a meta-analysis.
Data synthesis	15a	NaN
	15b	NaN
	15c	NaN
	15d	Both narrative and tabular summary will be used to present evidence of the relationship of health-related event and outcome and examining the association between them. We do not plan to conduct a meta-analysis because we expect high heterogeneity of data measurement tools across country.<br>Narrative synthesis will be done following domains:<br>1) Developing a preliminary synthesis of findings of included studies<br>2) Exploring relationships within and between studies<br>a) Developing a theory explaining the role of hypoxia as a aetiological risk factor for neurodevelopmental disorders in the form of DLD depending on the DLD subtype (dyslexia, dysorthography, reading and math problems);<br>b) Assessing if the presence of hypoxia in a group of children with DLD increases the risk of comorbidity of other neurodevelopmental disorders.<br>c) Assessing if the occurrence of perinatal hypoxia increases the risk of DLD and DLD with comorbid neurodevelopmental disorders depending on the sex of the child.<br>3) Assessing the robustness of the synthesis<br>Additionally, if it is possible to collect sufficient information, we will analyze the following subgroups:<br>– children with a history of prenatal/perinatal hypoxia caused by various factors;<br>– children with different types of DLD;<br>– children with different levels of severity of DLD.
Meta-bias(es)	16	NaN

Confidence in cumulative evidence	17	The Narrative Synthesis (NS) guidelines will allow us to transparently report the data from the included studies. We will use the tools and techniques for assessing robustness of the synthesis described in the Guidance on the Conduct of Narrative Synthesis in Systematic Reviews. This will allow you to assess the quality of the collected data.

*** It is strongly recommended that this checklist be read in conjunction with the PRISMA-P Explanation and Elaboration (cite when available) for important clarification on the items. Amendments to a review protocol should be tracked and dated.** **The copyright for PRISMA-P (including checklist) is held by the PRISMA-P Group and is distributed under a Creative Commons Attribution Licence 4.0.**

From: Shamseer L, Moher D, Clarke M, Ghersi D, Liberati A, Petticrew M, Shekelle P, Stewart L, PRISMA-P Group. Preferred reporting items for systematic review and meta-analysis protocols (PRISMA-P) 2015: elaboration and explanation. BMJ. 2015 Jan 2;349(jan02 1):g7647.
